# Healing of induced tongue defects using erythropoietin hydrogel (an experimental study on rats)

**DOI:** 10.1186/s12903-024-04161-5

**Published:** 2024-03-27

**Authors:** Fatma Ata, Rana El-Qashty, Meran Farid, Jilan Youssef

**Affiliations:** 1 Oral Medicine, Periodontology, Diagnosis and Oral Radiology department, Faculty of Dentistry, Mansoura University, Mansoura, Egypt; 2 Oral Biology department, Faculty of Dentistry, Mansoura University, Mansoura, Egypt; 3 Periodontology and Oral Medicine department, Faculty of Dentistry, Horus University, Demiatta, Egypt

**Keywords:** Anti-inducible nitric oxide synthase, Anti-vascular endothelial growth factor, Tongue ulcer, Connective tissue, Therapeutic strategy

## Abstract

**Background:**

Tongue is complex muscular organ that may be affected by recurrent or chronic ulcerations and malignances that require effective treatment to enhance healing and tissue regeneration. So, this study aimed to evaluate the efficiency of erythropoietin (EPO) hydrogel as an anti-inflammatory and an inducer of neovascularization during healing of induced rats’ tongue defects.

**Methods:**

Thirty six rats were divided into three groups; Group I (negative control): tongues were left without ulceration and received no treatment, Group II (positive control): tongue defects were prepared on the tongues’ dorsal surfaces, measuring (5 mm × 2 mm) using a tissue punch rotary drill for standardization, and left untreated, Group III (EPO group): tongue defects were prepared as in group II, then injected circumferentially around wound margins with a single high dose of EPO hydrogel of 5000 U/kg on the day of defect preparation. Animals were euthanized on seventh and fourteenth days after treatment, tongue specimens were collected, and paraffin blocks were prepared and processed for histological assessment by hematoxylin and eosin stain and immunohistochemical evaluation of anti-iNOS and anti-VEGF followed by histomorphometrical analysis and the relevant statistical tests.

**Results:**

At both time points, the EPO treated group showed significantly enhanced tissue regeneration marked by the histologically better regenerated tissue with well developed, thick walled and well-organized blood vessels and significant reduction in defect depth compared to positive control group. EPO group also showed significant decrease in iNOS and significant increase in VEGF antibodies indicating its anti-inflammatory and neovascularization effects respectively.

**Conclusion:**

EPO treatment can significantly accelerate regeneration and filling of tongue defects by reducing tissue inflammation and enhancing neovascularization. Therefore, EPO could be a potential therapeutic strategy for accelerating healing of tongue ulcers. However, further investigations are required to optimize the dose and unravel any potential side effects before its clinical application.

## Background

The oral and maxillofacial region is responsible for several vital activities including speech, mastication, swallowing and appearance where patients’ overall health is largely affected by their oral health [1]. Tongue is a complex muscular organ that is used for mastication, swallowing, and speech. However, it can be affected by a variety of diseases and neoplasms including erythroplakia, granular cell tumor, squamous cell carcinoma, and kaposi sarcoma which might damage it and necessitate surgical excision of the tongue’s affected area [2, 3].

Oral ulcers can also affect the tongue with several possible etiologies. Depending on their characteristics and course, they can be either acute or chronic [4]. All of these lesions negatively impact the patients’ sociality, physical and psychological health. Hence, management of these oral disorders is of critical relevance to enhance patients’ quality of life [1].

Treatment of oral and maxillofacial diseases could possibly include antibiotics, analgesics [5], anti-inflammatories, angiogenic factors [6], herbal medicines [7], and specific regional treatments, such as chemical cauterization, surgical removal, and laser therapy [8]. However, these treatment may not cause significant improvement or can even result in several unexpected side effects [9].

The management of oral disorders involves the use of conventional drug-delivery system (DDS) that mainly involves tablets, oral gels, and lozenges for defect repair through the application of suitable biomaterials [10]. However, this type of treatment is quite challenging due to the distinctiveness of the oral environment as well as the oral and maxillofacial structures complexity [11]. Furthermore, the increased moistness of the oral cavity combined with tongue movement complicates the maintenance of consistent medicinal patch control at the injury site hindering treatment of oral mucosal diseases [12].

Consequently, creating an on-demand and convenient DDS turns out to be extremely important. Hydrogels have shown distinct structural and functional characteristics that set them apart from other biomaterials. Hydrogels have demonstrated encouraging potential to both promote structural defect repair and have an effective therapeutic effect at the lesion sites [13].

Hydrogels are biological materials formulated through chemical or physical monomers crosslinking reactions, developing a polymer network system [14]. They are distinguished by their capacity to hold large volumes of water or other bio-liquids, as well as their stable three-dimensional structure, but they do not themselves cure oral disorders or correct faults. They serve as an excellent carrier or platform that transmit different components such as medications, cells, and inorganic minerals and provide space and microenvironments to support the operation of the loaded components. They have demonstrated significant promises and possibilities in the fields of biomedicine since their beginnings [15].

Through in-situ osmotic administration, hydrogels can encapsulate medications and other therapeutic substances and transport them to the affected site, producing long-lasting and efficient therapeutic effects [16]. Furthermore, hydrogels can be used in tissue regenerative engineering to encapsulate and culture a variety of stem cells or cytokines because of their biocompatibility and structure, which are comparable to that of natural extracellular matrix [17].

Erythropoietin is a 34 kDa glycoprotein hormone and a member of the hematopoietic class I cytokine superfamily. It regulates red blood cell count by inducing the proliferation and differentiation of precursor cells and preventing the apoptosis of bone marrow erythroid cells [18]. It has been established that cells other than hematopoietic cells express the EPO receptor such as the nervous system, through its astrocytes, neurons, and vascular endothelial brain cells [19] as well as the cardiovascular system, through its cardiomyocytes, endothelial cells, and circulating precursor cells that represent the main nonhematopoietic EPO targets [20],

Numerous nonhematopoietic effects of erythropoietin have been reported enhancing the healing following tissue damage through inhibition of the inflammatory cytokines’ actions, and prevention of programmed cell death [21]. Additionally, erythropoietin exhibits proangiogenic and cytoprotective properties by antagonizing and modifying pro-inflammatory cytokines such as tumor necrosis factor-alpha (TNF-α) controlling excessive inflammation [22]. The oral mucosa’s basal cells also exhibit erythropoietin receptors [23]. Therefore, it is anticipated that EPO application either topically or systemically could be effective in healing oral lesions.

Nitric oxide (NO) is one of the most valuable and broadly investigated free radical molecules. It has a significant role regulating physiological processes, immunity, and antagonizing inflammation under physiological circumstances. However, it has a cytotoxic effect causing tissue damage in case of overexpression [24]. Nitric oxide synthase (NOS) is an enzyme responsible for NO production. It has three isoforms: endothelial NOS (eNOS), neuronal NOS (nNOS), and inducible NOS (iNOS). Unlike the other two isoforms, iNOS is an inducible calcium-independent synthetic enzyme that is released in response to inflammation, producing much higher amounts of NO than the other two isoforms [25].

The primary regulator for neovascularization is the vascular endothelial growth factor (VEGF). It regulates differentiation and growth enhancing vascular permeability, and anti-apoptosis. Moreover, it is critical for the development and establishment of new blood vessels and lymph systems [26]. It belongs to the endothelial growth factor family that until now consists of seven members all contributing to various vascular systems: VEGF (A-F), and placenta growth factor [27].

VEGF interacts with specific receptors (VEGF-R) to achieve its biological purpose. These receptors are part of the receptor tyrosine kinase (RTK) subfamily [28] that represent one of the most significant signaling pathways that orchestrate angiogenesis [29]. The attachment of VEGF to its receptor activates specific proteins that signal the endothelial cell’s nucleus inducing secretion of molecules required for development of new endothelial cells [30].

Animal models help in assessment of various disorders. The physiological body functions of rats as mammalians have been found to be nearly similar to those of human beings. Moreover, rats’ immune system can survive the induction of many diseases [31]. So, the aim of the present study was to evaluate the effectiveness of erythropoietin hydrogel in alleviating inflammation and inducing neovascularization during healing of induced rats’ tongue defects.

## Materials and methods

### Animals

Thirty-six adult, healthy, Sprague Dawley, male rats with weight ranging between 250 and 300 g were used in the study. The rats were housed in cages (20 cm × 40 cm), 3 rats/cage, in the medical experimental research center (MERC) (Mansoura University, Mansoura, Egypt), at a room with controlled temperature of 26°C, relative humidity of 65–70% and on a 12-h light–dark cycle with access to water ad libitum and commercial diet. They were accustomed for at least 2 weeks before the start of the study.

### Surgical procedures of mechanical tongue defect

The present study was performed in accordance with ARRIVE guidelines. Before any surgical intervention, the weight of each rat was recorded for adjusting different drugs administration doses. Anesthetization was performed by intramuscular injection of xylazine hydrochloride (5–7 mg/kg) and ketamine hydrochloride (35–45 mg/kg). Prior to defect preparation, rats’ tongues were wiped with Betadine. Then, defects were prepared according to our previous protocol [32] in the middle thirds’ median line on the tongues’ dorsal surfaces, measuring (5 mm × 2 mm) using a tissue punch rotary drill (cat. #4159, IQ implants USA, Maryland, USA). The bases of the punched tissues were incised using surgical scissors. For three days postoperatively, oxytetracycline hydrochloride 20% and analgin 0.5 mg were administered daily to rats through intraperitoneal injection.

### Preparation and characterization of erythropoietin loaded CS/β-GP/gelatin hydrogel

Erythropoietin hydrogel was prepared according to the protocol described by Xu et al. [33]. Briefly, 40 mg of chitosan nanoparticles (CS-NPs) were dissolved in 20 mL of 0.1% acetic acid solution under stirring. Next, 5 mg of gelatin were dissolved in 1 mL of deionized water at room temperature and the resulting solutions were filtered through a 0.22 μm syringe filter. Subsequently, 1.02 g of β-sodium glycerophosphate (β-GP) were dissolved in 2.8 mL of 0.1% (W/V) tripolyphosphate (TPP) solution and filtered through a 0.22 μm syringe filter, after which 2.21 mL of EPO (ATC code: B03XA01, Janssen-Cilag Ltd, High Wycombe, Bucks, UK, 10,000 U/mL) were added. After forming a uniform mixture of the prepared solutions, 0.1 mol/L sodium hydroxide (NaOH) solution was added in drops into the mixture to adjust the pH to 7.0. The EPO loaded CS/β-GP/Gelatin hydrogel was assembled through incubation of the mixture for 5 min at 37 °C.

The particle size analyzer Dynamic Light Scattering (DLS) (Zetasizer Nano ZN, Malvern Panalytical Ltd, United Kingdom) was used for analysis of particle size and size distribution in the form of the average volume diameters and polydispersity index by photon correlation spectroscopy at fixed angle of 173° at 25° C. Samples were investigated in triplicate. The particle size was found to be 345.5 ± 13.01 nm. Zeta potential was determined using the same equipment which was found to be 12.1 ± 0.87 mV.

### Study design

Sample size was calculated based on defect depth mean among studied groups retrieved from previous research [32]. Using G*power program version 3.1.9.4 to calculate sample size based on effect size of 2.5153, 2-tailed test, power = 90.0% and α error = 0.05 then the appropriate sample size was found to be at least five in each group. So, six rats were used in each subgroup to counterbalance any rats’ drop rate.

This was a randomized controlled, experimental study. Using simple random sampling method, thirty-six rats were divided into three main groups (12 rats each) as follows: ***Group I (negative control group)***: The tongues were left without ulceration and received no treatment. ***Group II (positive control group)***: The tongue defects were prepared, then left untreated to heal normally. ***Group III (EPO group)***: The tongue defects were prepared, then injected circumferentially around wound margins with a single high dose of erythropoietin hydrogel of 5000 U/kg on the day of defect preparation [34].

Six rats from each group were euthanized by anesthesia overdose (≥ 0.86 mg/kg sodium pentobarbital intraperitoneal) on the 7^th^ and the remaining rats were euthanized on 14^th^ day after defect preparation. Tongue samples were collected and processed for histological and immunohistochemical examination. The carcass was wrapped in plastic bags and carefully transported to be burnt in an incinerator.

### Histological and immunohistochemical staining

After neutral-buffered formalin fixation, paraffin blocks of tongue specimens were prepared, then 4 μm serial tissue sections were cut using a microtome. Deparaffinization, rehydration, then hematoxylin and eosin staining (H&E) were performed for assessment of changes in defect depth healing progress, and tissue regeneration. For immunohistochemical staining, H_2_O_2_ was used for blocking endogenous peroxidase, then antigens were retrieved through boiling in citrate buffer. Slides were then incubated with the primary antibodies for inducible nitric oxide synthase (iNOS) (Rabbit recombinant multiclonal [RM1017] to iNOS, cat. #ab283655, Abcam, Cambridge, UK, dilution 1:2000) as an inflammatory marker and vascular endothelial growth factor (VEGF) (Rabbit monoclonal [Y103] to VEGF Receptor 1, cat. # ab32152, Abcam, Cambridge, UK, dilution 1:250) to assess neovascularization followed by incubation with the secondary biotinylated antibody, then streptavidin biotin complex. Diaminobenzidine chromogen (DAB substrate kit, cat. # ab64238, Abcam, Cambridge, UK) was applied followed by counterstaining with Harris hematoxylin.

### Digital image analysis

H&E slides were visualized and photographed using ToupCam® digital camera (model no. XCAM1080PHA) attached to Olympus®, CX22, Japan, light microscope with 0.5 photo adaptor, using 4x objective lens. For iNOS and VEGF immunohistochemically stained sections, five different sites (1 × 1 mm^2^) in each slide were evaluated by two blinded examiners using a 10x objective lens and photographed. Image processing software Fiji ImageJ (version 2; NIH, Maryland, USA) was used for digital image analysis where the vertical defect depth was measured for each slide and the percentage of positive brown staining area regardless of stain intensity to the total area was calculated.

For the defect depth, the Fiji ImageJ measuring function after image calibration was used. For staining surface area, the method described by Patera et al. [35] was modified. Briefly, the function color deconvolution 2 (histological dyes digital separation) was applied to the microphotographs providing three independent digital images (H&E, DAB, and a complementary image), after these stain-specific values were determined. Data was presented as the mean vertical depth or positive brown staining percentage ± standard deviation.

### Statistical analysis

Data analysis was done using GraphPad Prism 9 (GraphPad Software). The normality of data was tested using Shapiro-Wilk test. Quantitative data was presented as mean ± standard deviation for normally distributed data. The obtained results’ significance was assessed at the 0.05 level. The two-way ANOVA test was used to evaluate the combined effect of grouping and time independent factors on the dependent continuous outcomes which were defect depth, iNOS, and VEGF antibody immunostaining using Post Hoc Tukey test for pairwise comparison.

## Results

### Hematoxylin and Eosin (H & E) histological staining results

**Fig. 1:**
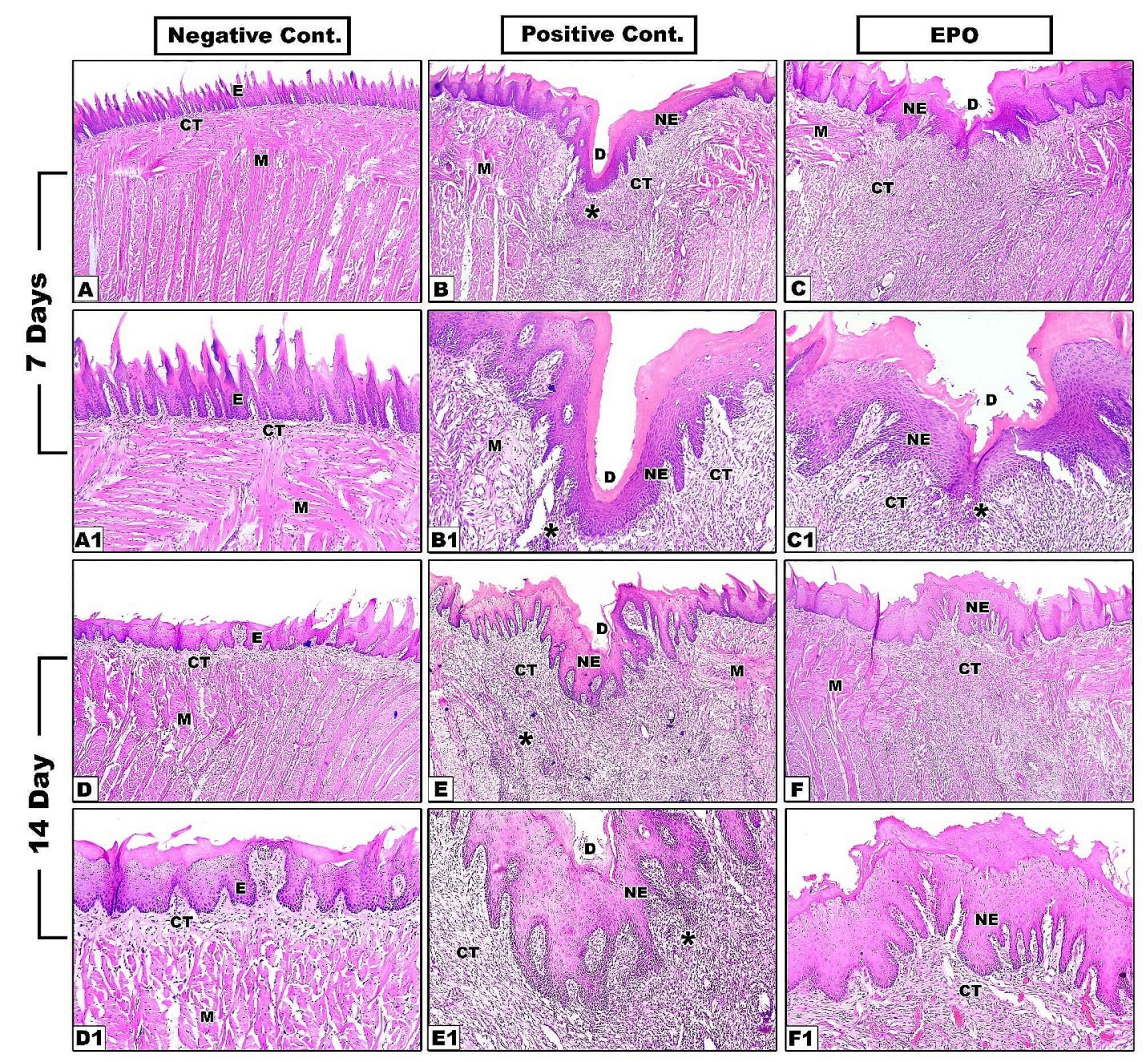
Hematoxylin and Eosin staining results of tongue specimens of different groups at 7^th^ and 14^th^ days (**A-F x4**), (**A1-F1 x10**). E: epithelium, NE: new epithelium, CT: connective tissue, M: muscles, D: defect, Asterisk: inflammatory infiltrate

Histological assessment of the normal tongue specimens from negative control group at both 7^th^ and 14^th^ day timepoints revealed the existence of keratinized epithelium of normal regular thickness showing well-organized regular, tapered, conically shaped filliform lingual papillae with normal mushroom shaped fungiform papillae carrying normal taste buds scattered between them. The underlying connective tissue (CT) showed regular CT papillae interdigitations with the overlying epithelium and well-organized tongue musculature with normal horizontal and vertical orientation **(**Fig. 1A, A1, D, D1).

On the seventh day, the positive control group showed deep defects covered by keratinized epithelium that didn’t contain the characteristic lingual papillae. The underling CT was heavily infiltrated by numerous inflammatory cells with no signs of muscle regeneration **(**Fig. 1B, B1). In EPO treated group, shallower defects also covered by keratinized epithelium without lingual papillae were observed. The underlying CT was less intensely infiltrated by inflammatory cells, signs of new blood vessels, and more organized collagen fibers, but no muscles regeneration **(**Fig. 1C, C1).

**Fig. 2:**
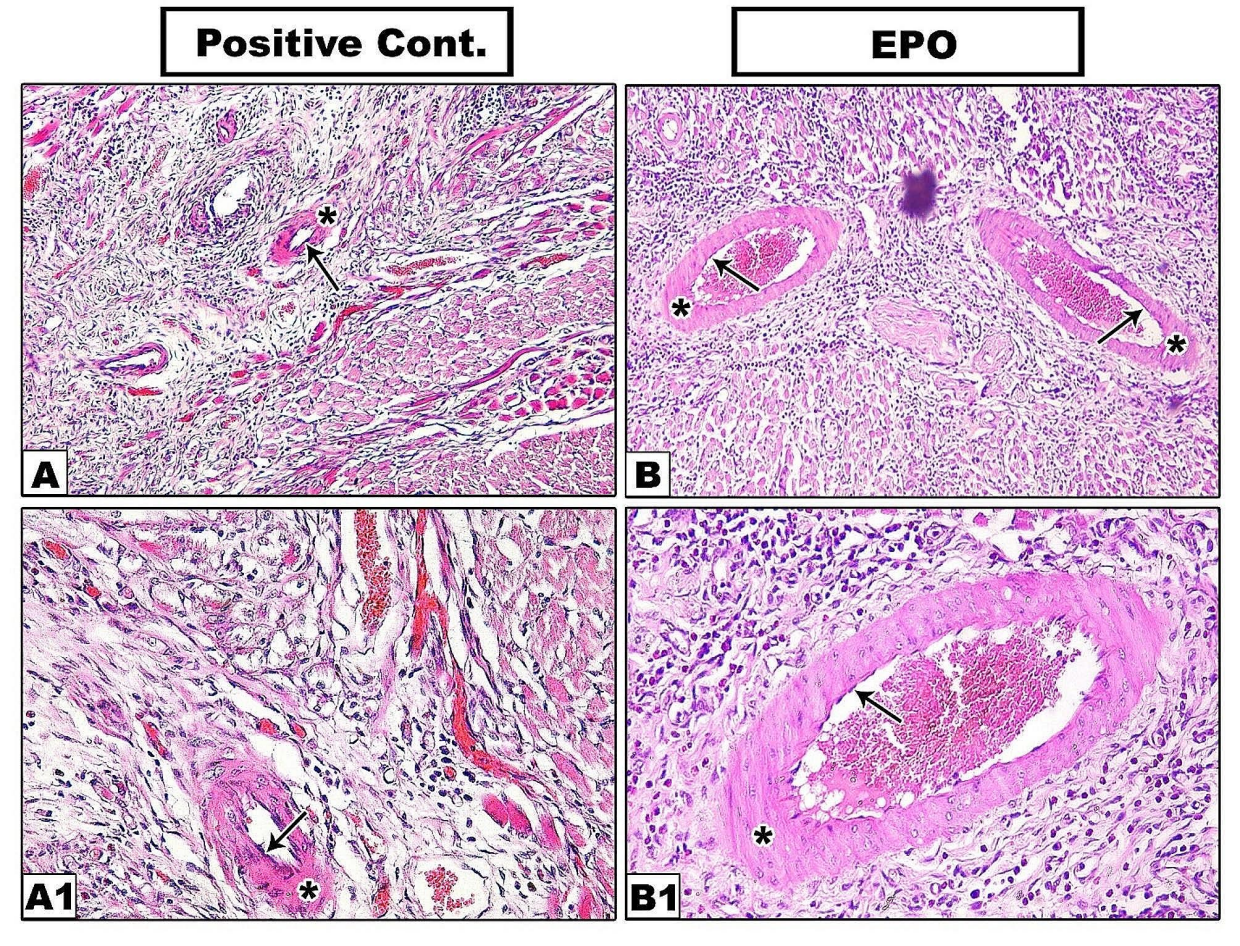
Hematoxylin and Eosin staining results showing newly formed blood vessels in positive control (**A, A1**) and EPO-treated group (**B, B1**) on the 14^th^ day. Arrow: tunica intima, Asterisk: tunica media

On the fourteenth day, the defects in the positive control group were still relatively deep, covered by keratinized epithelium without lingual papillae. The underlying connective tissue showed small newly regenerated well organized blood vessels without restoration of tongue musculature **(**Fig. 1E, E1). While in EPO group, defects were completely filled by newly regenerated tissue, covered by well-organized keratinized epithelium with numerous interdigitations with underlying CT. However, the typical lingual papillae were still not restored. The underlying CT showed well-arranged collagen bundles with newly formed muscle fibers **(**Fig. 1F, F1). It also showed enhanced neovascularization where the newly formed blood vessels were larger, well-developed, thick-walled with well-organized tunica media and tunica intima compared to those of positive control group (Fig. 2).

### Defect depth statistical analysis results

**Fig. 3:**
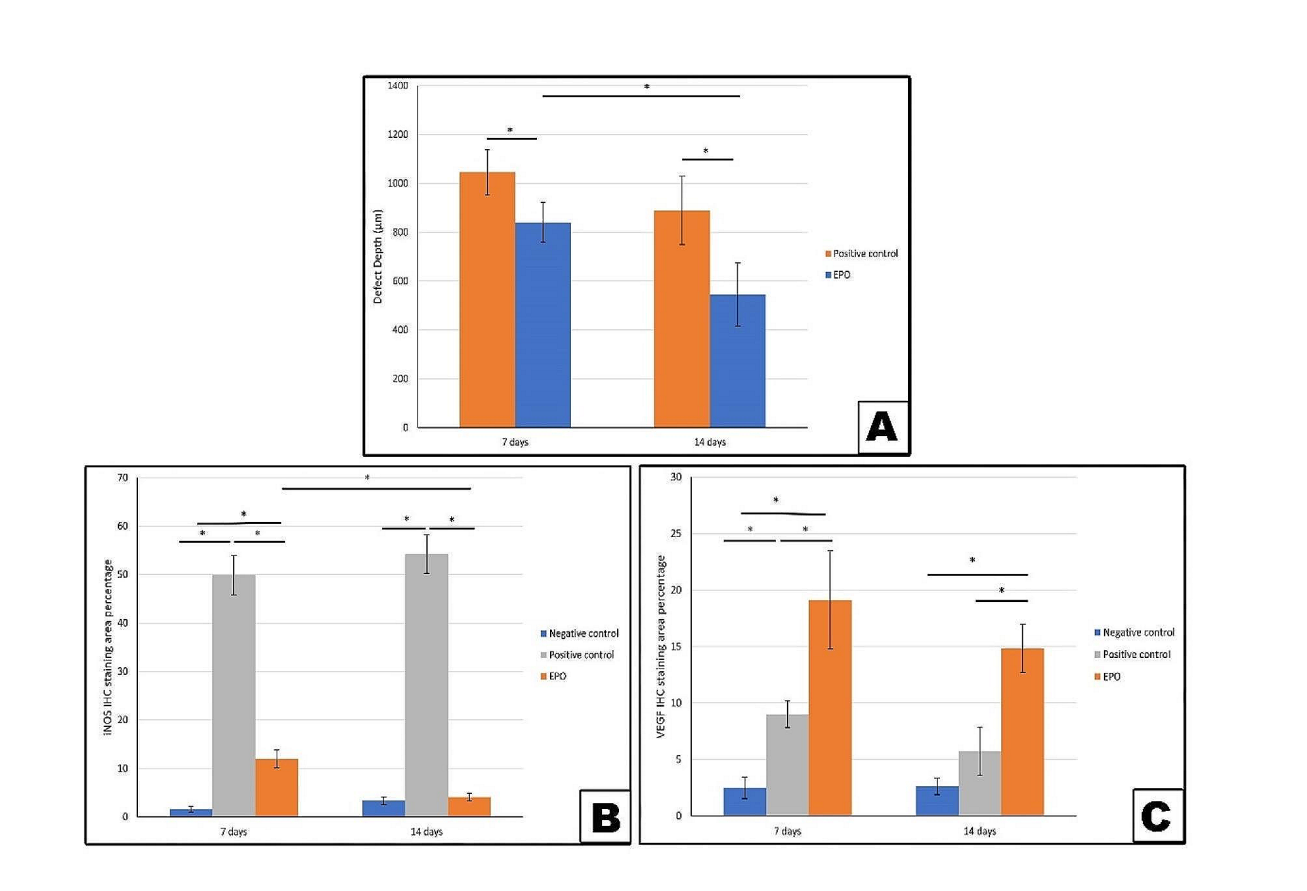
**A-C.** Bar graphs showing the two-way ANOVA statistical analysis for defect depth, anti-iNOS and anti-VEGF immunohistochemical staining results. * Denotes statistical significance

**Table 1:** Post Hoc Tukey test for pairwise comparison of factors affecting defect depth

Time of assessment	Positive control	EPO
7 days	1046.26 ± 92.70	839.85 ± 81.21^*^
14 days	889.55 ± 139.65	545.89 ± 130.16^*#^

*: Significant difference between groups within the same time point, #: Significant difference between time points within the same group at *p* < 0.05.

**Table 2:** Two-way ANOVA for prediction of combined effect of changing groups and time of assessment on defect depth

Source	Type III Sum of Squares	df	Mean Square	F	*p* value.
Grouping	378,222	1	378,222	29.30	< 0.0001*
Time assessment	253,881	1	253,881	19.67	0.0004*
Grouping * time assessment	23,549	1	23,549	1.824	*P* = 0.1956
Error	206,527	16	12,908		
Total	862,178	19			

Df: degree of freedom *P*: Probability *: significance < 0.05.

Post Hoc Tukey test for pairwise comparison of defect depth revealed significant decrease in EPO treated group compared to positive control group at both timepoints. Unlike the positive control group that showed a non-significant depth decrease in 14^th^ day group compared to 7^th^ day group, EPO treated group showed significant depth decrease (Fig. 3A**)**, (Table 1). Two-way ANOVA showed significant effect of time alone and of grouping alone, but non-significant effect of their interaction (*P* < 0.05) (Table 2).

### Immunohistochemical staining results

#### Inducible nitric oxide synthase (iNOS) antibody

**Fig. 4:**
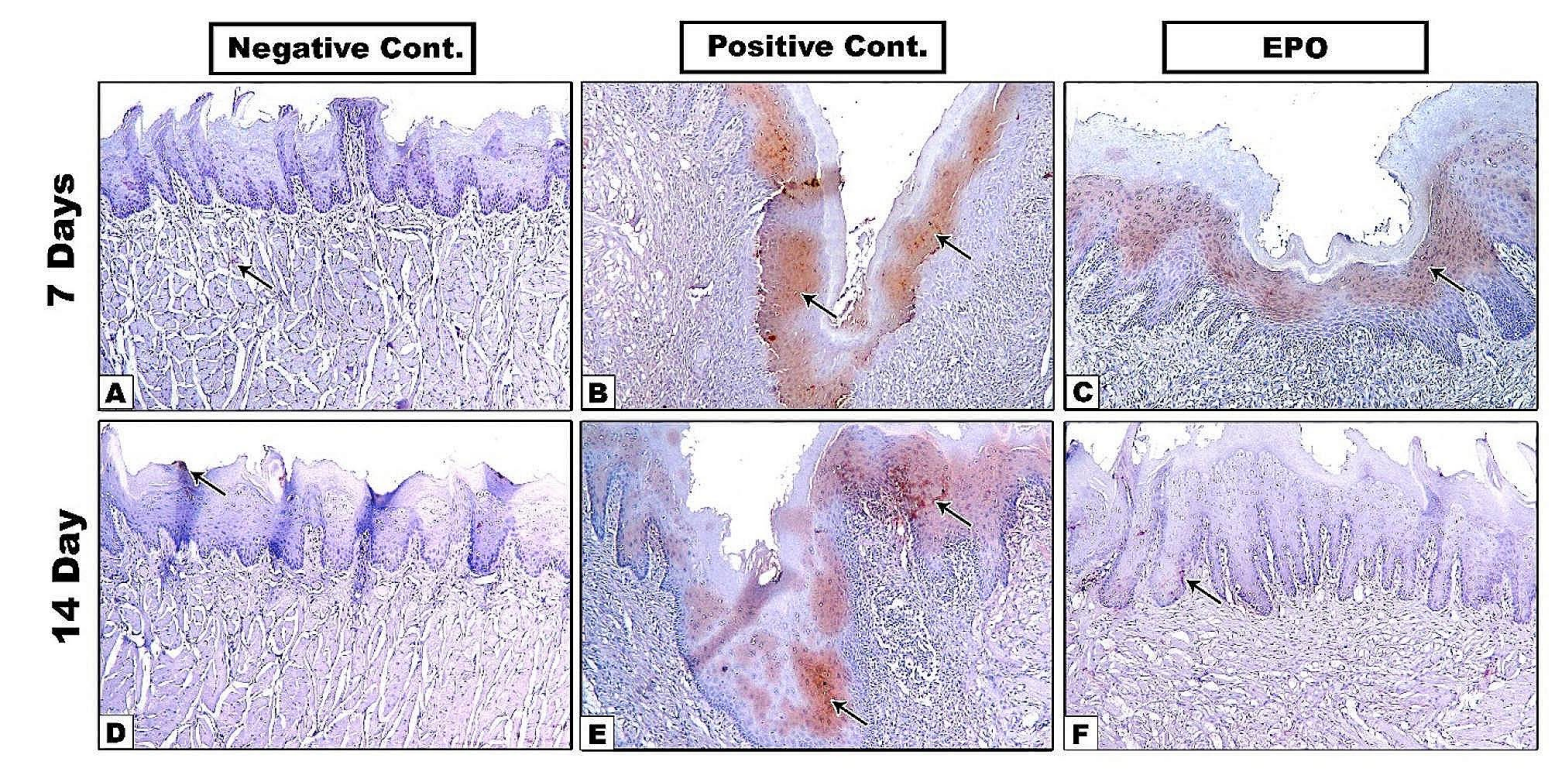
**A-F.** Immunostaining for iNOS antibody. Arrow: positive brown staining areas

**Table 3:** Post Hoc Tukey test for pairwise comparison of factors affecting iNOS IHC positive staining surface area percentage

Time of assessment	Negative control	Positive control	EPO
7 days	1.57 ± 0.62	49.89 ± 4.07^#^	11.98 ± 1.88^#+^
14 days	3.34 ± 0.78	54.20 ± 3.96^#^	4.09 ± 0.74^*+^

^*^Significance vs. 7 days group within the same intervention. ^#^ Significance vs. negative control group, and ^+^ significance vs. positive control group within the same time point.

**Table 4:** Two-way ANOVA for prediction of combined effect of changing groups and time of assessment on iNOS IHC positive staining surface area percentage

Source	Type III Sum of Squares	df	Mean Square	F	*p* value
Grouping	14,808	2	7404	F (2, 24) = 17.00	*P* < 0.0001*
Time	2.005	1	2.005	F (1, 24) = 0.3238	*P* = 0.5746
Grouping * time assessment	210.5	2	105.3	F (2, 24) = 1196	*P* < 0.0001*
Error	148.6	24	6.193		
Corrected Total	15,169	29			

Df: degree of freedom P: Probability *: significance < 0.05

As shown in Fig. 4, the anti-iNOS positive immunostaining appeared as dark brown cytoplasmic stain in the epithelial basal and suprabasal cell layers. In Fig. 3B, the bar graph of iNOS immunostaining results for the positive control 14^th^ day subgroup showed a non-significant increase relative to the 7^th^ day subgroup. However, both subgroups had significantly higher levels of inflammation compared to other groups at all time periods.

On the other hand, the EPO treated 14^th^ day subgroup showed a significant decrease relative to the 7^th^ day one and both subgroups showed significant decrease compared to the positive control subgroups at both timepoints. The EPO treated group also revealed a significant increase on the 7^th^ day and a non-significant increase on the 14^th^ day compared to the negative control group. (Table 3). The two-way ANOVA revealed significant effect of grouping, and grouping-by-time interaction, but non-significant effect of time factor (*P* < 0.05) (Table 4).

#### Vascular endothelial growth factor (VEGF) antibody

**Fig. 5:**
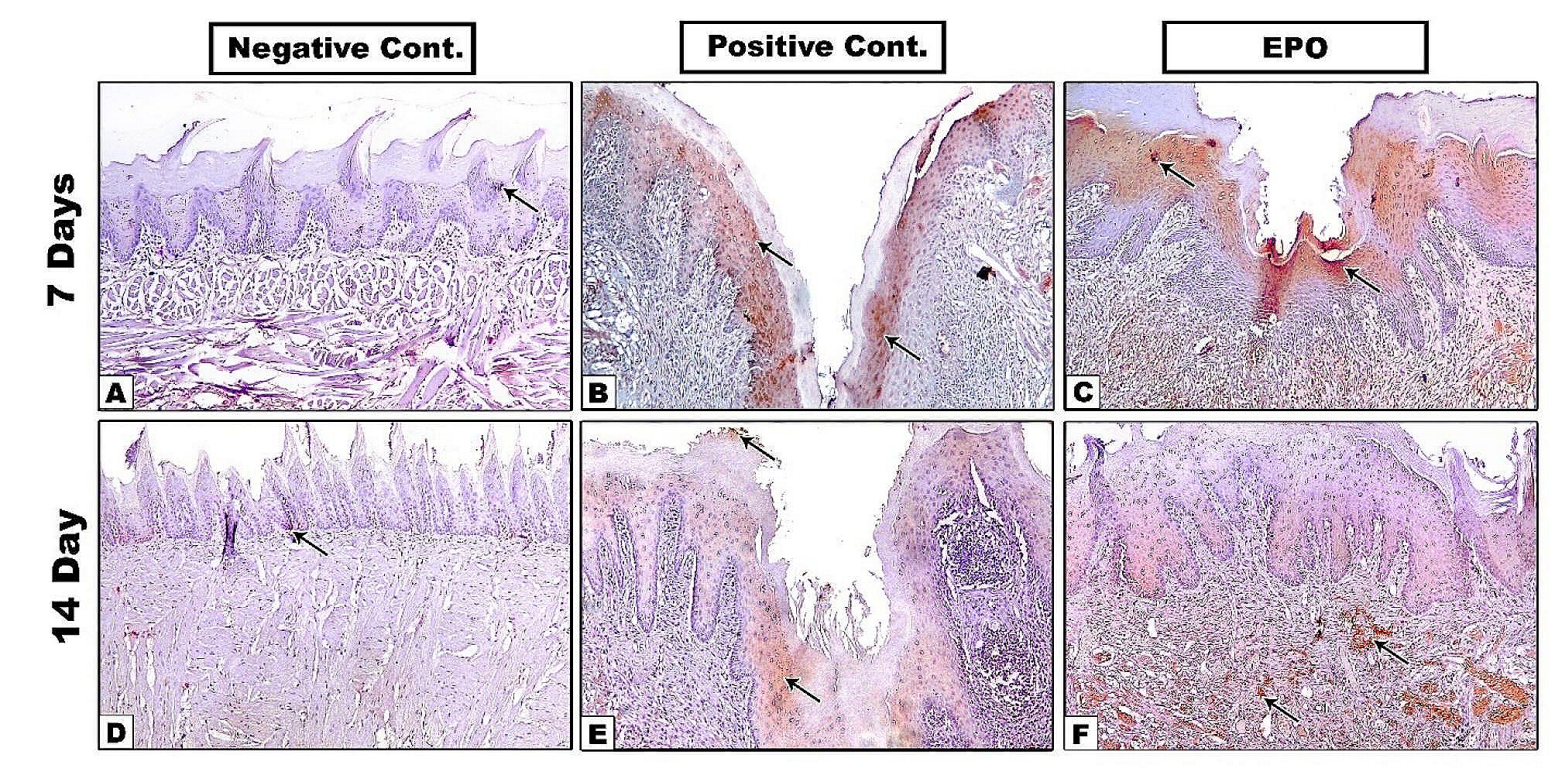
**A-F.** Immunostaining for VEGF antibody. Arrow: positive brown staining areas

**Table 5:** Post Hoc Tukey test for pairwise comparison of factors affecting VEGF IHC positive staining surface area percentage

Time of assessment	Negative control	Positive control	EPO
7 days	2.49 ± 0.95	9.01 ± 1.19^#^	19.13 ± 4.37^#+^
14 days	2.63 ± 0.75	5.73 ± 1.80	14.83 ± 2.13^#+^

^#^ Significance vs. negative control group, ^+^ significance vs. positive control group within the same time point.

**Table 6:** Two-way ANOVA for prediction of combined effect of changing groups and time of assessment on VEGF IHC positive staining surface area percentage

Source	Type III Sum of Squares	df	Mean Square	F	*p* value
Grouping	2	1078	538.9	F (2, 24) = 2.729	*P* = 0.0855
Time	1	46.23	46.23	F (1, 24) = 9.347	*P* = 0.0054*
Grouping * time assessment	2	27	13.5	F (2, 24) = 109.0	*P* < 0.0001*
Error	24	118.7	4.946		
Corrected Total	29	1270			

Df: degree of freedom P: Probability *: significance < 0.05.

The anti-VEGF immunostaining positive reaction results appeared as brown cytoplasmic deposits in the epithelial basal and suprabasal cell layers as well as in the lamina propria (Fig. 5**)**. Compared to the negative control group, VEGF positive immunostaining was significantly increased in the positive control group on the 7^th^ day, but non-significantly increased on the 14^th^ day. The wound healing progress and vascularization as measured by VEGF was also significantly higher in the EPO treated group compared to other groups at both timepoints. **(**Fig. 3C), (Table 5). The two-way ANOVA showed a non-significant effect of grouping and significant effect of time, and grouping-by-time interaction (*P* < 0.05) (Table 6).

## Discussion

Oral ulcers represent the most common widespread, debilitating disorders affecting oral cavity and dramatically affecting the quality of involved patients’ lives [36]. Eventhough most oral ulcers can self-heal due to good vascularization of oral tissues, cases with recurrent, severe, or chronic ulceration usually require additional treatment modalities to restore the physiology and function of the affected tissue. These treatments include the application of steroids, analgesics, tetracycline, and antiseptic mouthwashes [37]. However, the prolonged use of these medicines has various side effects, including acne, sweating, rashes, elevated blood glucose levels and weight gain. Steroids also can affect bones and worsen the current infections or increase incidence of new infections [38]. So, the aim of the current study was to evaluate erythropoietin hydrogel as a possible treatment modality to enhance healing of tongue ulcers and assess its anti-inflammatory and neovascularization effects.

Erythropoietin can enhance wound healing through four main mechanisms. First, EPO has an anti-apoptotic effect through the inhibition of inflammatory cells activity and suppression of the proinflammatory cytokines production as interleukins IL-6, IL-1β, TNF-α, membrane lipid peroxidase and reactive oxygen species [39]. Second, it induces a more efficient tissue repair through granulation tissue formation in the wound healing earlier stages and its remodeling at subsequent stages which could be attributed to the decreased wound content of inflammatory cells [40]. Third, it improves angiogenesis through formation of new functioning capillaries activated by VEGF that has a synergistic effect with EPO [39, 41]. Fourth, it increases erythrocytes and leukocytes number within the defect improving tissue oxygenation and accelerating cell growth [42].

In the present study, the hydrogel was formulated using chitosan (CS) and β-sodium glycerophosphate (β-GP) for their good biocompatibility and ability to construct a hydrogel at body temperature [43]. The gelatin was incorporated to crosslink CS and β-GP electrostatically through interaction between anions and cations to lessen the gelation period [44]. All of these criteria allowed the formulation of hydrogel that provided a sustained erythropoietin release [45].

Eventhough nitric oxide (NO) under normal physiological conditions exerts an anti-inflammatory effect, it was found to have a pro-inflammatory effect in abnormal situations [46] as the other pro-inflammatory cytokines causes overexpression of the iNOS in granulocytes, monocyte/ macrophages, neutrophil and many other cells. As a result, substantial amounts of NO are released, exceeding the physiological NO levels by up to one thousand folds [47]. This fact was consistent with the present study results where even in negative control group, there was positive expression of iNOS antibody.

The positive iNOS antibody immunostaining showed significant increase in positive control group compared to negative control at all time points indicating inflammatory reaction associated with tongue defect preparation. Also, EPO treated group showed significant decrease in 14^th^ day subgroup compared to the 7^th^ day one and both subgroups revealed significant decrease compared to the positive control group at both timepoints confirming time-dependent anti-inflammatory effect associated with EPO treatment.

In accordance with our results, Kandasamy et al. [48] reported that EPO successfully attenuated the iNOS mRNA expression and NO overproduction in a mouse sepsis model. Also, in study conducted by Huang et al. [49] EPO successfully ameliorated inflammation in systemic lupus erythematosus mice through suppression of iNOS expression and regulation of macrophage inflammatory reaction. Mohamed et al. [50] also confirmed the protective effect of EPO against acute renal injury induced by cisplatin through iNOS formation inhibition.

Vascular endothelial growth factor (VEGF) is a glycoprotein known to increase vascular permeability, induce vascular endothelial cells growth, and promote activation and chemotaxis of monocytes/macrophages. It is implicated in neovascularization by increasing microvasculature and therefore accelerating wound healing process [51]. These facts were consistent with the present study findings where EPO treated group showed significant increase in VEGF levels which was associated with formation of numerous, more organized, and thick-walled blood vessels that was reflected in enhanced wound healing and more rapid filling of defect compared to positive control group.

Similarly, Heitrich et al. [52] found that EPO treatment attenuated the damaging effect of sepsis on kidneys and lung through the VEGF overexpression. According to Javadmoosavi et al. [42] EPO treatment significantly reduced the inflammatory cells density, induced neovascularization which was attributed to the increased VEGF levels improving wound tissue oxygenation and nourishment, increased the number of fibroblasts and thickness of epithelium at the wound margins. Also, in a study conducted by Bakhshi et al. [53] EPO treatment initiated VEGF release that had a positive effect on healing of tibiofibular fracture and osteogenesis.

Our findings were also consistent with Yaghobee et al. [54] who informed that topical EPO treatment enhanced the healing of surgical palatal wounds which achieved significantly completed epithelialization relative to the control group. In a study conducted by Bader et al. [55] EPO-Hydrogel treatment of animals’ deep-dermal scalds resulted in much faster healing, earlier wound re-epithelialization, accelerated extracellular matrix maturation, enhanced angiogenesis with numerous capillaries, which was confirmed by elevated VEGF and CD31 levels. In a further work, Bader et al. [56] also found that erythropoietin significantly accelerated the wound epithelialization and healing as early as seven days postoperatively following topical application in both acute and chronic wounds. Several other experimental studies also confirmed the efficacy of EPO in treating dermal ischemic or diabetic lesions when applied topically or systemically [57, 58].

In review by Günter G. [59], he concluded that the recombinant human erythropoietin successfully improved the wound healing process through its anti-inflammatory effect, and by increasing the capillary density in the ischemic flaps promoting enhanced healing in both earlier and later phases of injury repair. Similarly in another study conducted by Ahn et al. [60] EPO treatment during the acute phase of hindlimb ischemia significantly enhanced blood flow and angiogenesis.

The clinical trial conducted by Yaghobee et al. [61] confirmed that EPO topical application can speed up the gingival grafts healing which was associated by decrease in the inflammatory reaction during healing period. Tobalem et al. [62] also informed a dose-dependent positive effect of systemic EPO early treatment reducing inflammation and accelerating healing of burn wounds. Toleubayev et al. [63] also stated a positive effect of EPO in several wound healing models, for instance traumatized wounds, infected wounds, chronic ischemic wounds, and diabetic ones where EPO significantly reduced tissue inflammation and accelerated the blood vessels regeneration.

On the other hand, Aoshiba et al. [64] evaluated EPO administration in a murine model of endotoxin shock with a dose of 1000 IU/Kg and found that despite the decrease in the mortality rate, there was no apparent modifications in the inflammatory response. Also, in another study conducted by Arslantaş et al. [65] that evaluated the effect of systemic erythropoietin on healing of rats’ ischemic wounds reported that in EPO treated group, tissue flaps showed ulceration, necrosis, and abscess formation eventhough it had a positive effect on the 7^th^ day, also two rats in the EPO group deceased owing to tissue necrosis and infection which could be attributed to the elevated hematocrit and hemoglobin levels, hindering microcirculation. Takano et al. [66] also reported that eventhough EPO successfully reduced the apoptosis of lymphoid tissue, it didn’t enhance the survival rate in experimental sepsis model.

This difference in results could be accredited to the type of used EPO as certain types such as carbamylated EPO or some EPO mutants fail to bind to the classical EPO receptor and therefore don’t have any hematopoiesis in human cells. EPO could have either a tissue-protecting effect through anti-apoptosis, neuroprotection, cyto-protection, or potentially unfavorable effects through thromboembolism, extreme erythropoietic effect, and impaired microcirculation [59]. There are many other factors that could explain the difference in results between different studies such as the difference in route and dose of drug administration and the nature of the tissue and disease to be treated. As a limitation of the present study, only one dose of EPO was chosen based on previous research. However, in future studies comparisons could be made between different doses to optimize the conditions with longer follow-up periods before its application in clinical trials.

## Conclusion

In conclusion, EPO treatment can significantly accelerate regeneration and filling of tongue defects by reducing tissue inflammation as confirmed by the decrease in iNOS levels supporting its anti-inflammatory effect and enhancing neovascularization as indicated by elevated VEGF levels. Therefore, EPO represents a potential therapy for accelerating healing of tongue ulcers.

## Data Availability

All the produced data is integrated in this article.

## Abbreviations

EPO: Erythropoietin
iNOS: Inducible nitric oxide synthase
VEGF: Vascular endothelial growth factor
DDS: Drug-delivery system
TNF-α: Tumor necrosis factor-alpha
NO: Nitric oxide
NOS: Nitric oxide synthase
eNOS: Endothelial nitric oxide synthase
nNOS: Neuronal nitric oxide synthase
RTK: Receptor tyrosine kinase subfamily
CS-NPs: Chitosan nanoparticles
β-GP: β-sodium glycerophosphate
TPP: Tripolyphosphate
NaOH: Sodium hydroxide solution
DLS: Dynamic Light Scattering
H&E: Hematoxylin and eosin staining
ANOVA: Analysis of variance
CT: Connective tissue
MU-ACUC: Mansoura University animal care and use committee
